# Fasting Plasma Glucose as Initial Screening for Diabetes and Prediabetes in Irish Adults: The Diabetes Mellitus and Vascular Health Initiative (DMVhi)

**DOI:** 10.1371/journal.pone.0122704

**Published:** 2015-04-15

**Authors:** Margaret Sinnott, Brendan T. Kinsley, Abaigeal D. Jackson, Cathal Walsh, Tony O’Grady, John J. Nolan, Peter Gaffney, Gerard Boran, Cecily Kelleher, Bernadette Carr

**Affiliations:** 1 Vhi Healthcare Ltd, Dublin, Republic of Ireland; 2 Mater Misericordiae University Hospital, Dublin, Republic of Ireland; 3 Department of Statistics, Trinity College Dublin, Dublin, Republic of Ireland; 4 Steno Diabetes Center, DK-2820 Gentofte, Denmark; 5 The Adelaide and Meath Hospital incorporating the National Children’s Hospital, Dublin, Republic of Ireland; 6 University College Dublin School of Public Health, Physiotherapy and Population Science, University College Dublin, Dublin, Republic of Ireland; Johns Hopkins Bloomberg School of Public Health, UNITED STATES

## Abstract

**Objective:**

Type 2 diabetes has a long pre clinical asymptomatic phase. Early detection may delay or arrest disease progression. The Diabetes Mellitus and Vascular health initiative (DMVhi) was initiated as a prospective longitudinal cohort study on the prevalence of undiagnosed Type 2 diabetes and prediabetes, diabetes risk and cardiovascular risk in a cohort of Irish adults aged 45-75 years.

**Research Design and Methods:**

Members of the largest Irish private health insurance provider aged 45 to 75 years were invited to participate in the study. Exclusion criteria: already diagnosed with diabetes or taking oral hypoglycaemic agents. Participants completed a detailed medical questionnaire, had weight, height, waist and hip circumference and blood pressure measured. Fasting blood samples were taken for fasting plasma glucose (FPG). Those with FPG in the impaired fasting glucose (IFG) range had a 75gm oral glucose tolerance test performed.

**Results:**

122,531 subjects were invited to participate. 29,144 (24%) completed the study. The prevalence of undiagnosed diabetes was 1.8%, of impaired fasting glucose (IFG) was 7.1% and of impaired glucose tolerance (IGT) was 2.9%. Dysglycaemia increased among those aged 45-54, 55-64 and 65-75 years in both males (10.6%, 18.5%, 21.7% respectively) and females (4.3%, 8.6%, 10.9% respectively). Undiagnosed T2D, IFG and IGT were all associated with gender, age, blood pressure, BMI, abdominal obesity, family history of diabetes and triglyceride levels. Using FPG as initial screening may underestimate the prevalence of T2D in the study population.

**Conclusions:**

This study is the largest screening study for diabetes and prediabetes in the Irish population. Follow up of this cohort will provide data on progression to diabetes and on cardiovascular outcomes.

## Introduction

The increasing incidence of type 2 diabetes worldwide is a major health concern. In 2013, the worldwide prevalence of diabetes had reached 382 million (8.3%) people [1]. Type 2 diabetes has a long asymptomatic pre-clinical phase during which time 20–30% of patients develop complications such as retinopathy, cardiovascular disease, neuropathy and nephropathy [2, 3]. Early detection followed by lifestyle modification and/or pharmacotherapy can delay or arrest disease progression [4, 5].

Knowledge of local disease burden and epidemiology is required for targeting healthcare interventions [6, 7]. Direct estimation by following a large, population-based cohort is an optimal approach for monitoring population prevalence [8, 9]. Prevalence estimates from regional studies can be extrapolated for the entire nation, though the accuracy of estimates derived from small local studies is questionable especially if the study population was pre-selected for recognised type 2 diabetes risk factors [7,10]. National prevalence data on undiagnosed type 2 diabetes, local information on the distribution of risk factors among type 2 diabetes and prediabetes populations and prognostic information on the risk of developing type 2 diabetes is important in health care planning [11, 12].

The Diabetes Mellitus and Vascular health initiative (DMVhi) study commenced in 2009 as a prospective longitudinal cohort study to provide information on the prevalence of type 2 diabetes, prediabetes, diabetes risk and cardiovascular risk in a large cohort of Irish adults aged 45–75 years. In this study, we sought to estimate prevalence of undiagnosed type 2 diabetes and prediabetes in the cohort using a two-step screening strategy; fasting plasma glucose (FPG) as the initial screening test followed by an oral glucose tolerance test (OGTT) for subjects with impaired fasting glucose (IFG). Use of FPG as initial screening may underestimate the prevalence of diabetes in the study population. However given the size of the DMVhi cohort it was not possible to perform OGTT on the entire population.

## Research Design and Methods

### Study participants

Healthcare in Ireland is provided through a mixed public-private system. The DMVhi cohort was recruited from a list of private health insurance policy holders with Vhi Healthcare. This private healthcare insurer covers 54% of the Irish private health market (approximately 26% of the Irish population). Study inclusion criteria were based on age of the named policy holder (45–75 years) and, initially (in 2009), residence in the catchment area of either of two Dublin University Teaching Hospitals. The geographical area was then extended incrementally over the following three years to include all of counties Cork (population 519,032) and Dublin (population 1,273069). Exclusion criteria were: already diagnosed with diabetes or prescribed oral hypoglycaemic agents or insulin. Between January 2009 and December 2012, 122,531 eligible policy holders were issued a letter inviting them to attend a health assessment and 29,548 agreed to participate (24.1%). The Joint Ethics Committee of St. James’ Hospital and the Adelaide and Meath National Children’s Hospital, Dublin approved the study protocol. All participants signed informed consent.

### Health assessment

Participants were invited to attend a health assessment following a minimum fasting period of 8 hours. Health assessments involved the completion of a detailed medical questionnaire, anthropometric measurements and blood tests, which were undertaken at Vhi Medical Centres by trained clinical staff. The medical questionnaire gathered data on demographics, lifestyle, personal and family medical history. Smoking status was categorised as smoker, non-smoker or ex-smoker, and frequency of exercise was defined as performing an activity for ≥30 minutes everyday, either <1 day/week, 1–4 days/week or ≥5 days/week. Alcohol and daily vegetable/fruit consumption was recorded. A medical history of cardiovascular disease was defined as having angina, myocardial infarction, bypass surgery/stenting, stroke or transient ischaemic attack. A family history of diabetes was defined as having either immediate family members or other relatives with diabetes. Risk of developing diabetes was assessed using the modified FINDRISC scoring system [13].

Blood pressure (mm Hg), height (cm), weight (kg), waist and hip circumference (cm) and BMI (kg/m^2^) were measured. Abdominal obesity was defined as a waist circumference of ≥94cm for males and ≥80cm for females as per the International Diabetes Federation’s definition of the metabolic syndrome for Europids [14].

Two fasting venous blood samples were taken; one for plasma glucose, the other for lipid profile analysis. Blood samples were transferred on the same day to an accredited laboratory and analysed on the ROCHE diagnostics modular platform [15]. Fasting total cholesterol, high density lipoproteins (HDL) and triglycerides were measured and an indirect measurement of low density lipoprotein (LDL) was determined using the Friedewald equation for those with serum triglycerides ≤4.0 mmol/L [16].

Glucose tolerance categories (type 2 diabetes, impaired fasting glucose and Impaired Glucose Tolerance) were defined according to ADA guidelines [5]. Both the participant and their primary physician received a copy of the final medical report. Follow-up of individuals with type 2 diabetes, impaired fasting glucose, impaired glucose tolerance or other significantly abnormal results was undertaken by the primary physician. The initial blood glucose assessment was based on venous fasting plasma glucose (FPG). An FPG <5.6mmol/L was normal; FPG ≥5.6≤6.9mmol/L was IFG and FPG ≥7.0mmol/L was in the diabetes range. Participants with an FPG ≥7.0 mmol/L were diagnosed with diabetes if symptoms were present; otherwise the FPG was repeated on another day to confirm the diagnosis. Subjects with a FPG in the IFG range underwent 75g oral glucose tolerance testing (OGTT) to further assess for impaired fasting glucose, impaired glucose tolerance and diabetes in this subgroup.

### Statistical analysis

Prevalence rates and 95% confidence intervals (CIs) for undiagnosed type 2 diabetes, impaired fasting glucose and impaired glucose tolerance were calculated for the entire DMVhi population and subgroups according to age-group, gender and other covariates. Prevalence rates and patient characteristics were compared across glucose tolerance categories using chi-squared tests and ANOVA. Crude and adjusted prevalence estimates (and 95% CIs) were derived for the national population. Estimates were adjusted for age-group (45–54, 55–64, 65–75 years) and gender distribution of the national population, which were defined using the 2011 census data from the Central Statistics Office of Ireland. The direct adjustment method of standardisation was applied. A multinomial logistic regression model was developed to examine the association of covariates with the odds of having undiagnosed T2D, IFG and IGT compared with normoglycaemia. Continuous variables: age; BMI; systolic blood pressure (BP); fasting lipid profile (cholesterol, HDL and LDL cholesterol, triglycerides) and categorical variables: gender; smoking; alcohol and daily vegetable/fruit consumption; exercise; abdominal obesity; family history of diabetes; medical history of cardiovascular disease and receipt of anti-hypertensive medication were included in a main effects model. The backward stepwise elimination method was applied and variables significant at a *p*-value of <0.05 were retained in the final model. Missing data underwent list-wise deletion during model development. Odds ratios (ORs) were fully adjusted for all covariates. Statistical analyses were performed using SPSS 20.0 for Windows (SPSS Inc, Chicago, Illinois).

## Results

29,548 subjects attended for screening with 404 (1.4%) failing to complete the screening protocol. A total of 29,144 individuals were included in the analysis; 32.2% were aged 45–54 years, 38.6% aged 55–64 years and 29.2% aged 65–75 years. Males accounted for 44.4% of the study population (Table 1).

**Table 1:** DMVhi cohort age-group and gender-specific prevalence rates of undiagnosed type 2 diabetes, impaired glucose tolerance and impaired fasting glucose (n = 29,144; male = 12,929 (44%); female = 16,215 (56%)).

Rates of T2D, IFG and IGT in the DMVhi Cohort						
		n	T2D	IFG	IGT	Normal
**All ages**		29144	1.8%	7.1%	2.9%	88.1%
	**Male**	12929	2.9%	10.0%	4.2%	83.0%
	**Female**	16215	1.0%	4.9%	1.9%	92.2%
**45–54**		9387	0.8%	4.8%	1.3%	93.0%
	**Male**	4016	1.3%	7.2%	2.1%	89.4%
	**Female**	5371	0.5%	3.1%	0.7%	95.7%
**55–64**		11259	1.9%	7.1%	3.1%	87.1%
	**Male**	4917	3.0%	11.1%	4.4%	81.5%
	**Female**	6342	1.0%	5.5%	2.1%	91.4%
**65–75**	**All**	8498	2.9%	2.5%	4.4%	84.0%
	**Male**	3996	4.4%	11.4%	5.9%	78.3%
	**Female**	**4502**	1.7%	6.1%	3.1%	89.1%

T2D = type 2 diabetes, IGT = impaired glucose tolerance and IFG = impaired fasting glucose. Gender differences for DMVhi cohort and for each age group: p<0.001

### Cohort prevalence of Dysglycaemia

**Fig 1:**
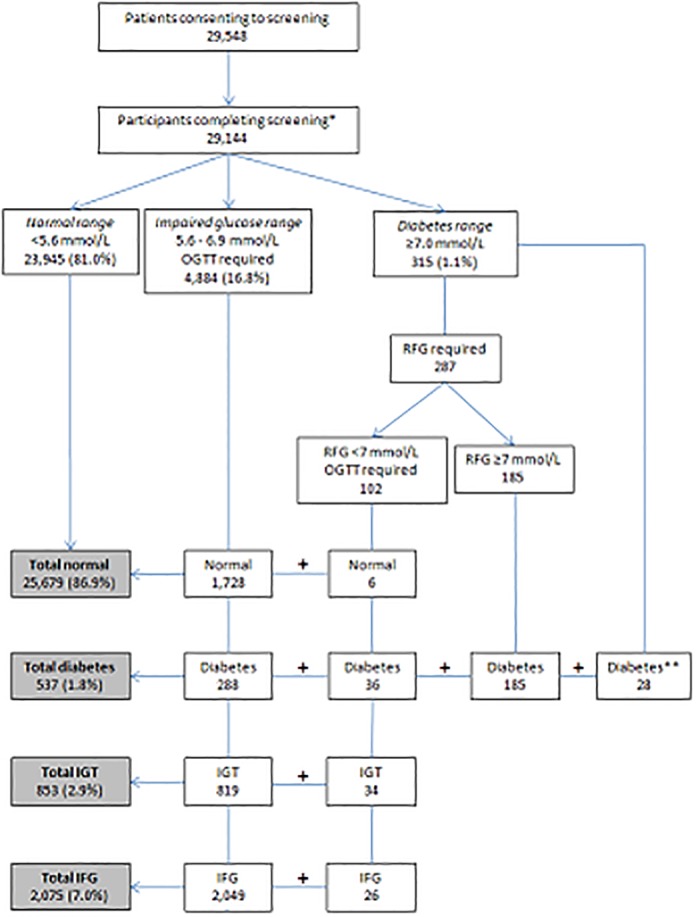
Results of DMVhi screening for undiagnosed type 2 diabetes, impaired fasting glucose and impaired glucose tolerance (March 2009-December 2012). RFG = repeat fasting glucose, IGT = impaired glucose tolerance, IFG = impaired fasting glucose, OGTT = oral glucose tolerance test. *Participants were not deemed to have completed screening if they did not return for required OGTT/RFG (n = 322) or did not provide a venous fasting sample (n = 82). ** Diagnosed with diabetes on fasting blood glucose (≥7.0 mmol/L) and symptoms of diabetes.

Following completion of 29,144 screenings, 11.8% had abnormal glucose metabolism (i.e. undiagnosed type 2 diabetes confirmed in 1.8%; impaired fasting glucose confirmed in 7.1% and, of the 4884(16.8%) requiring an OGTT, impaired glucose tolerance was confirmed in 2.9%) (Fig. 1). As only the subgroup of the population with impaired fasting glucose had an OGTT performed, this value is not a true population prevalence for impaired glucose tolerance. In those aged 45–54, 55–64 and 65–75 years, rates of type 2 diabetes T2D were 0.8%, 1.9% and 2.9% respectively. IFG prevalence was greatest in the 55–64 year age-group (3.1% compared with 1.6% in those aged 45–54 and 2.5% in 65–75 year olds) (Table 1). Impaired glucose tolerance prevalence by age group, following initial FPG ≥5.6≤6.9mmol/L, was 1.3%, 3.1% and 4.4% for 45–54 years, 55–64 years and 65–75 years respectively.

#### Gender differences

The male to female composition of the DMVhi cohort (Table 1) is 44% and 56% respectively. Mean age for males was 59.5 ±8.1 years and 59.0 ±8.0 years for females, (p<0.001).

In males, 17.1% had abnormal plasma glucose levels compared with 7.8% for females. Prevalence rates in each diagnostic category were higher in males (2.9% type 2 diabetes, 10.0% impaired fasting glucose and 4.2% impaired glucose tolerance) than in females (1.0%, 4.9% and 1.9% respectively) (*p*<0.001). Across all age-gender groupings, the prevalence of type 2 diabetes, impaired fasting glucose and impaired glucose tolerance in males was 1.9–3.0 times higher than in females (Table 1). Males aged 65–75 years had the highest rate of type 2 diabetes (4.4%), impaired fasting glucose (11.4%) and impaired glucose tolerance (5.9%). Rates of impaired glucose tolerance were higher in males than females at all age groups (Table 1).

Compared with females, males had a higher BMI (27.5 vs. 26.1 kg/m^2^), higher systolic BP (126.4 vs. 123.3 mmHg), greater frequency of CVD (5.9% vs. 2.2%) and use of anti-hypertensive medication (22.7% vs. 18.9%). Also, more males than females consumed alcohol (82.9% vs. 77.3%) and smoked (current and ex-smokers) (47.4% vs. 34.0%). *P-* values were <0.001 for each of the parameters mentioned above.

#### National prevalence estimates

Based on our data, estimates of national prevalence of undiagnosed type 2 diabetes and impaired fasting glucose were determined following adjustment for age-group and gender in the national population. Study cohort prevalence rates were marginally higher than adjusted national prevalence rates: prevalence rates for type 2 diabetes were 1.80% versus 1.76% and 7.12% versus 7.04% for impaired fasting glucose respectively. Based on extrapolation of these figures to Irish adults aged 45–75 years (numbers based on Central Statistics Office of Ireland 2011 Census), 24,110 (95% confidence intervals (CIs): 23,723–24,571) were estimated to have undiagnosed type 2 diabetes and 96,484 (95% CIs: 89,989–103,571) to have impaired fasting glucose. National prevalence of impaired glucose tolerance was not estimated since all participants did not have an OGTT. Therefore a minimum of 120,594, 8.8% of the adjusted age-specific national population, individuals were estimated to have undiagnosed type 2 diabetes (1.76%) and impaired fasting glucose (7.04%).

Type 2 diabetes national standardised prevalence estimates were highest in the oldest age-group (65–75 years), and higher in males than females across all age-groups. National estimates of impaired fasting glucose in both males and females peaked in the middle age-group (55–64 years).

### Risk profiles for undiagnosed type 2 diabetes, impaired fasting glucose and impaired glucose tolerance

Table 2 shows participant characteristics by abnormal glucose category. Compared to normoglycaemic participants, those in any category of impaired glucose metabolism were more likely to be older, male, smokers (current and past), to have abdominal obesity, with a higher BMI, higher systolic BP, elevated triglycerides, lower HDL and LDL cholesterol, be receiving anti-hypertensive medication, have a history of cardiovascular disease (CVD), and have a family history of diabetes (all *p*-values <0.001). They were also less likely to exercise ≥5 days/week (*p*<0.001) and eat vegetables/fruit everyday (*p* = 0.01).

**Table 2:** DMVhi study participant characteristics by glucose tolerance status (n = 29,144).

Characteristics	Normal glucose (±SD or %)	Undiagnosed T2D (±SD or %)	IGT (±SD or %)	IFG (±SD or %)	p value
**n**	25679	537 (1.8%)	853 (2.9%)	2075 (7.1%)	
**Male**	10730 (41.8%)	372 (69.3%)	540 (63.3%)	1287 (62.0%)	<0.001
Age* (years)	58.9 (±8.1)	62.8 (±7.0)	63.1 (±7.5)	61.0 (±7.5)	<0.001
**Ever smoked**	9928 (38.7%)	283 (52.7%)	404 (47.4%)	1033 (49.8%)	<0.001
**Alcohol consumption**	20404 (79.5%)	408 (76.0%)	680 (79.7%)	1753 (84.5%)	<0.001
**Exercise ≥5 days/week**	9326 (36.3%)	147 (27.4%)	226 (26.5%)	644 (31.0%)	<0.001
BMI* (kg/m ^**2**^ **)**	26.3 (±4.2)	30.9 (±5.0)	29.8 (±4.3)	28.8 (±4.3)	<0.001
**Abdominal obesity** ^**§**^	16036 (62.4%)	484 (90.1%)	754 (88.4%)	1698 (81.8%)	<0.001
Systolic BP* (mm/Hg)	123.6 (±16.8)	135.6 (±17.0)	135.0 (±16.3)	130.5 (±17.0)	<0.001
Total cholesterol* (mmol/L)	5.3 (±0.9)	5.1 (±1.0)	5.2 (±1.0)	5.3 (±1.0)	<0.001
LDL cholesterol* (mmol/L)	3.2 (±0.9)	3.0 (±0.9)	3.1 (±0.9)	3.1 (±0.9)	<0.001
HDL cholesterol* (mmol/L)	1.7 (±0.5)	1.3 (±0.4)	1.4 (±0.4)	1.5 (±0.4)	<0.001
Triglycerides* (mmol/L)	1.1 (±0.6)	1.8 (±1.0)	1.6 (±0.9)	1.4 (±0.8)	<0.001
**Anti-hypertensive medication**	5996 (20.6%)	244 (45.4%)	371 (43.5%)	619 (29.8%)	<0.001
**Cardio-vascular disease**	1119 (3.8%)	45 (8.4%)	57 (6.7%)	138 (6.7%)	<0.001
**Family history of diabetes**	7973 (31.0%)	248 (46.2%)	314 (36.8%)	766 (36.9%)	<0.001

*Mean value,

^§^ abdominal obesity defined as waist circumference of ≥80cm in females and ≥94cm in males. BP = blood pressure, T2D = type 2 diabetes,

Odds ratios of association with undiagnosed diabetes, impaired fasting glucose and impaired glucose tolerance compared with having normal glucose levels were determined for lifestyle and metabolic factors (Table 3). Eight risk factors (gender, age, systolic BP, receipt of anti-hypertensive medication, a family history of diabetes, abdominal obesity, BMI and triglycerides) were associated with significantly higher odds of being in an abnormal glucose category. Risk factors that had the strongest independent association with undiagnosed type 2 diabetes were having a family history of diabetes (OR: 2.2, 95% CIs: 1.9–2.7) and being male (OR: 2.1, 95% CIs: 1.7–2.6). The odds of having undiagnosed type 2 diabetes was increased by 89% for every 5kg/m^2^ increase in BMI and by 83% for every ten-year increase in age (years). The risk factor with the highest odds of association with impaired fasting glucose was being male (OR: 1.9, 95% CIs 1.7–2.1), while the risk factor with the strongest independent association with impaired glucose tolerance was age (OR: 1.9, 95% CIs: 1.7–2.1).

**Table 3:** Multivariable-adjusted odds ratios and 95% confidence intervals for having undiagnosed T2D, impaired glucose tolerance and impaired fasting glucose (compared with normoglycaemia) in the DMVhi study cohort.

	Undiagnosed T2D	IGT	IFG
	**Odds Ratios (95% CIs)**	**Odds Ratios (95% CIs)**	**Odds Ratios (95% CIs)**
**Male**	2.1 (1.7–2.6)	1.7 (1.4–2.0)	1.9 (1.7–2.1)
**Age (per 10 years)**	1.8 (1.6–2.1)	1.9 (1.7–2.1)	1.4 (1.3–1.5)
**Systolic BP (10 mm Hg)**	1.2 (1.1–1.3)	1.2 (1.1–1.3)	1.1 (1.07–1.14)
**Anti-hypertensive medication**	1.5 (1.2–1.8)	1.5 (1.3–1.8)	1.1 (1.0–1.3)
**Family history of diabetes**	2.2 (1.9–2.7)	1.4 (1.2–1.7)	1.5 (1.3–1.6)
**Abdominal obesity** ^**§**^	1.6 (1.1–2.2)	1.8 (1.4–2.3)	1.5 (1.3–1.7)
**BMI (per 5kg/m** ^**2**^ **)**	1.9 (1.7–2.1)	1.7 (1.5–1.8)	1.5 (1.4–1.6)
**Alcohol consumption**	1.0 (0.8–1.3)*	1.3 (1.1–1.6)	1.4 (1.3–1.6)
**Triglycerides (per 0.1 mmol/L)**	1.12 (1.1–1.13)	1.08 (1.07–1.1)	1.04 (1.03–1.05)
**LDL cholesterol (per 1 mmol/L)**	1.8 (1.3–2.6)	1.7 (1.3–2.3)	1.0 (0.9–1.2)*
**Total cholesterol (per 1 mmol/L)**	0.5 (0.3–0.7)	0.5 (0.4–0.7)	0.9 (0.8–1.1)*

^§^ abdominal obesity defined as waist circumference of ≥80cm in females and ≥94cm in males,

*NS = p value not significant (p>0.05). T2D = type 2 diabetes, IGT = impaired glucose tolerance, IFG = impaired fasting glucose, CIs = confidence intervals, BMI = body mass index

## Discussion

Results from the DMVhi cohort indicate that type 2 diabetes and prediabetes are important public health problems in the Republic of Ireland, with undetected abnormal glucose affecting at least 11.8% of adults aged 45–75 years. The rates of undiagnosed diabetes, impaired fasting glucose and impaired glucose tolerance were 1.8%, 7.1% and 2.9% respectively. The rate of undiagnosed diabetes increased with age from 0.8% in those aged 45–54 years to 2.95% in those aged 65–75 years. Those aged 55–64 years had the highest rate for impaired fasting glucose at 3.1% (Table 1). Initial screening in the DMVhi cohort was based on measurement of fasting plasma glucose, followed by an OGTT for participants whose FPG was in the IFG range, to further define the degree of abnormal glucose metabolism. Our results must be interpreted against this background, that initial screening using FPG may underestimate the prevalence of diabetes.

Early reports from the DECODE study identified that screening based on FPG underestimates undiagnosed diabetes and impaired glucose tolerance based on 2 hour post 75g glucose values [17]**.** Young and obese subjects are more likely to have diagnostic fasting glucose values than diagnostic 2 hour values [18]. Whilst for older subjects and females a greater proportion will have a diagnosis based on the 2-h glucose value [18].

The definition of impaired fasting glucose used in DECODE and other studies was a fasting plasma glucose between 6.1–6.9 mmol/l [17, 18]. In 2003 the ADA recommended that the lower fasting plasma glucose level for impaired fasting glucose be reduced to 5.6mmol/l. They commented that approximately 80% of the participants in the Diabetes Prevention Program with impaired glucose tolerance also had a fasting plasma glucose of ≥5.6≤6.9mmol/l [12]. In the DMVhi cohort 50% of those with impaired glucose tolerance and 12% with undiagnosed type 2 diabetes had an initial fasting plasma glucose in the 5.6–6.0mmol/L and would have been missed with the higher normal fasting plasma glucose cut-off. The definition of impaired fasting glucose in the DMVhi study improved detection of impaired glucose tolerance and type 2 diabetes compared to earlier reports with a fasting plasma glucose target of ≤6.1mmol/L.

Of those diagnosed with type 2 diabetes using OGTT, 7% (23 of 324) had a normal OGTT fasting glucose. Also, 25% of those diagnosed with impaired glucose tolerance had normal fasting glucose values on OGTT. However, we do not have data on the number of subjects in our cohort with an initial FPG below 5.6mmol/l who would have had either impaired glucose tolerance or type 2 diabetes based on a 2 hour post prandial value on OGTT. The proportion of missed cases of impaired glucose tolerance and type 2 diabetes must therefore be inferred from results of previous cohorts screened using OGTT. Vaccaro *et al*. performed OGTT on approximately 1300 healthy Italian subjects aged between 35–59 years and compared the prevalence of diabetes and prediabetes in subjects with impaired fasting glucose above 5.6mmol/l, as in the current DMVhi study [19]. They reported an impaired fasting glucose prevalence of 9.7% compared to our 7.1% (16.8% impaired fasting glucose based on one fasting plasma glucose. Using <5.6mmol/L as the normal fasting plasma glucose cut-off, Vaccaro *et al* also showed that 48.5% of those with impaired glucose tolerance and 18.7% of those with type 2 diabetes are missed if OGTT is not performed on subjects with a fasting plasma glucose below 5.6mmol/l.

Extrapolating these findings to our larger and older population would suggest that we have failed to identify 104 cases of type 2 diabetes (0.4% of the screened population) Therefore the corrected rate of type 2 diabetes in our population may be 2.2% (641). Using fasting plasma glucose as the initial screening test detected 853 (2.9%) cases of impaired glucose tolerance in our study. However this would represent only 51.5% of the likely rate based on Vaccaro *et al*, suggesting that an adjusted IGT rate of 5.6% (1632, or an additional 779 cases) in the DMVhi cohort.

Correcting the prevalence rates for cases missed as a result of our two step screening paradigm would result in an overall rate of undiagnosed type 2 diabetes of 2.2%, an impaired fasting glucose rate of 7.0% and an impaired glucose tolerance rate of 5.6% in the DMVhi cohort. The prevalence of undiagnosed type 2 diabetes and prediabetes in the DMVhi population could be as high as 16.3%. Correcting the national prevalence figures above for the under diagnosis of diabetes and impaired glucose tolerance as outlined would suggest that approximately 220,000 cases of undiagnosed diabetes and prediabetes may exist in the population of Irish adults aged 45–75 years.

The rate of undiagnosed type 2 diabetes in the DMVhi cohort (1.8%) is similar to the rate of 2.1% reported in a smaller Irish study of subjects selected from a general practice population by Smith *et al* [10]. Patients in this study were selected for screening based on risk factors for diabetes. In contrast DMVhi subjects were offered screening without risk factor based selection. Data from Janssen *et al*. in the ADDITION study on rates of undiagnosed diabetes in the Netherlands found a rate of 1% and a prediabetes rate of 1.8% in a relatively unselected European population [20]. Our data suggest a higher rate of undiagnosed diabetes and prediabetes in our study population. The dropout rate in the current study was considerably lower than the 24% reported by Janssen *et al* at 1.4% and, with no pre selection of subjects based on increased diabetes risk in our population, our results suggest a higher rate for undiagnosed type 2 diabetes and prediabetes in the DMVhi cohort.

The importance of accurate population data on the prevalence of type 2 diabetes is clear [6, 7]. To date there is no national surveillance programme, or national population-based survey of type 2 diabetes in Ireland. Though prevalence varies internationally, all available local data indicate that the prevalence of undiagnosed type 2 diabetes in Ireland may be lower than rates in Australia (3.7%) [21], United States (US) (2.8%) [22] and China (6.3%) [9]. Previous studies in Ireland have proposed rates of undiagnosed diabetes of 2.2% in 2003 and 2.7% in 2010 [10, 23]. However, as discussed above, subjects in these earlier studies were selected for the presence of risk factors and/or symptoms of T2D [10]. Similarly in the ADDITION study of subjects in general practice in three European countries, selection for screening for undiagnosed diabetes was based on achieving a threshold risk score following completion of a risk questionnaire [24]. In contrast, DMVhi participants were recruited from an unselected population invited to participate based on a letter of invitation only.

Data from the current study indicate that more males (2.9%) than females (1%) had undiagnosed type 2 diabetes and prediabetes (14.2% males, 6.8% females), consistent with results from previous studies on Irish cohorts [11, 23]. Undiagnosed type 2 diabetes has also been reported to occur with greater frequency in males in the US (3.6% males and 2.1% females) [22], and China (6.5% and 5.2% respectively) [9]. European cohort studies have shown that undiagnosed diabetes and impaired fasting glucose defined by isolated fasting hyperglycaemia, was more common in men than women between 30–69 years, whilst post load hyperglycaemia following OGTT was higher in women, particularly in the elderly population [25]. Data from the DECODE group showed that the proportion of previously undiagnosed diabetes was higher in men in young age groups and decreased with age above 70 years [25].

One of the strengths of the DMVhi study is that it is the largest assessment of metabolic and lifestyle determinants of undiagnosed type 2 diabetes prediabetes and CVD risk factors in Irish adults to date, comprising almost 30,000 subjects. National estimates derived using prevalence rates from large, multi-region cohort studies such as the DMVhi are likely to be more robust than previous estimates.

The potential weaknesses of the study must be considered. Non responders to the invitation to screening were not followed up, thus the study participants may not reflect the health status of the 122, 531 insured subjects initially invited. However the age and gender of study participants was similar to non-responders in this population.

The DMVhi cohort is 92% Caucasian European in ethnic origin and prevalence rates of abnormal glucose metabolism will reflect this. The cohort is drawn from those with private healthcare insurance from a mainly urban catchment area. Ownership of private healthcare insurance is likely a marker of higher socio-economic status and therefore higher socioeconomic groups may be over represented in our cohort compared to the national population (based on Central Statistics Office of Ireland population coded: 39% A, B, C1 (higher earners); 53% C2, D, E (lower earners). Vhi Healthcare members coded: 61% A, B, C1; 27% C2, D, E). As an inverse relationship between prevalence of type 2 diabetes and socio-economic status is recognised, national prevalence estimates based on the DMVhi cohort may underrepresent the true level of diabetes and prediabetes in the Irish population [26].

## Conclusions

Undiagnosed type 2 diabetes and prediabetes is a significant health issue for the population in the DMVhi cohort and, by extension, for this Irish population aged 45–75 years. The data from DMVhi suggests that unselected screening in this population results in a rate of undiagnosed type 2 diabetes and prediabetes which is higher than previous unselected populations in Europe and similar to rates seen in Irish populations selected based on risk factors for diabetes. This may be consistent with a higher rate of undiagnosed type 2 diabetes and prediabetes in the Irish population than was previously considered. Rates of type 2 diabetes and prediabetes in easily identifiable groups, such as older males, are significantly higher and suggest that targeted screening of high risk groups might be desirable. Given the gender differences in prevalence of type 2 diabetes and prediabetes seen in data from this and other cohorts (more common in males), and the data that more women than men are missed when screening using fasting plasma glucose, a screening paradigm using age and gender specific criteria could be considered. The DMVhi cohort data provides valuable information for strategic healthcare planning and focused use of healthcare resources.
